# Diffuse-Type Tenosynovial Giant Cell Tumor of the Knee with Concurrent Polymicrobial Infection (*Klebsiella oxytoca* and Group B *Streptococcus*)

**DOI:** 10.1155/2021/5523212

**Published:** 2021-08-14

**Authors:** J. Hunter Marshall, John G. Skedros, Chris F. Campana, Allan M. Seibert

**Affiliations:** ^1^Utah Orthopaedic Specialists and LDS Hospital, Salt Lake City, UT, USA; ^2^Intermountain Healthcare Department of Pathology, Salt Lake City, UT, USA; ^3^University of Utah Division of Infectious Diseases and Intermountain Healthcare Division of Infectious Diseases, Salt Lake City, UT, USA

## Abstract

Tenosynovial giant cell tumors (TGCT) are a rare class of benign proliferative tumors that are classified according to their presentation: localized-type (L-TGCT) or diffuse-type (D-TGCT). TGCT is synonymous with pigmented villonodular synovitis (PVNS). We describe the unique case of a 56-year-old obese male with type 2 diabetes who had polymicrobial septic arthritis of his left knee joint with concurrent D-TGCT in the same knee. While on a vacation, he noticed spontaneous left knee pain and swelling with an acute onset of fever. He was diagnosed with septic arthritis that was attributed to hematogenous spread from a leg laceration. The septic arthritis was treated with arthroscopic lavage and debridement, including simultaneous excision of the D-TGCT lesions, followed by intravenous ceftriaxone. Cultures of the synovial tissue that were obtained during arthroscopy grew *Klebsiella oxytoca* and beta-hemolytic (group B) *Streptococcus agalactiae.* We were not able to find another reported case of any joint with (1) a polymicrobial bacterial infection that included *Klebsiella oxytoca* and (2) concurrent bacterial septic arthritis and TGCT.

## 1. Introduction

Tenosynovial giant cell tumor (TGCT) is a general classification of rare, proliferative, usually benign, neoplastic lesion(s) most commonly affecting synovial membranes, tendons, and bursae of joints. TGCT is categorized into two subtypes: (1) localized type (a smooth encapsulated nodule) (L-TGCT) and (2) diffuse type (multiple nodules) (D-TGCT). TGCT is pathologically synonymous with pigmented villonodular synovitis (PVNS) and giant-cell tumor of the tendon sheath (GCTTS). Historically, the distinctions between PVNS, TGCT, and GCTTS were predicated on the anatomical location of the lesion(s) as being within the joint capsule, extra-articular regions, or with tendon sheath involvement [1]. TGCT has since become the standard classification for these diseases [2]. For this reason, we use D-TGCT to describe the pathologic diagnosis of our case.

D-TGCT has an incidence of approximately 5 to 8.4 new cases per million person-years [3, 4]. The most commonly affected joint is the knee, followed by the hip, ankle, shoulder, elbow, and digits in decreasing order [3–6]. The diagnosis of D-TGCT can be delayed for years because these lesions are generally slow growing and early symptoms resemble other conditions such as various forms of erosive arthritis, gout, and soft-tissue injuries. Our literature search (PubMed and Google Scholar) failed to reveal any case where a patient had both D-TGCT and septic arthritis of the same joint. We report a unique case of a type 2 diabetic patient who had D-TGCT with a coexisting polymicrobial bacterial infection of his left knee. The organisms grown in tissue culture from his knee included beta-hemolytic (group B) *Streptococcus agalactiae* (GBS) and *Klebsiella oxytoca* (KO), the latter being rarely involved in septic arthritis. We report this novel case in the perspective of an overview of the literature that deals with its microbiology, infection etiology, and pathology.

## 2. Case Presentation

**Figure 1:**
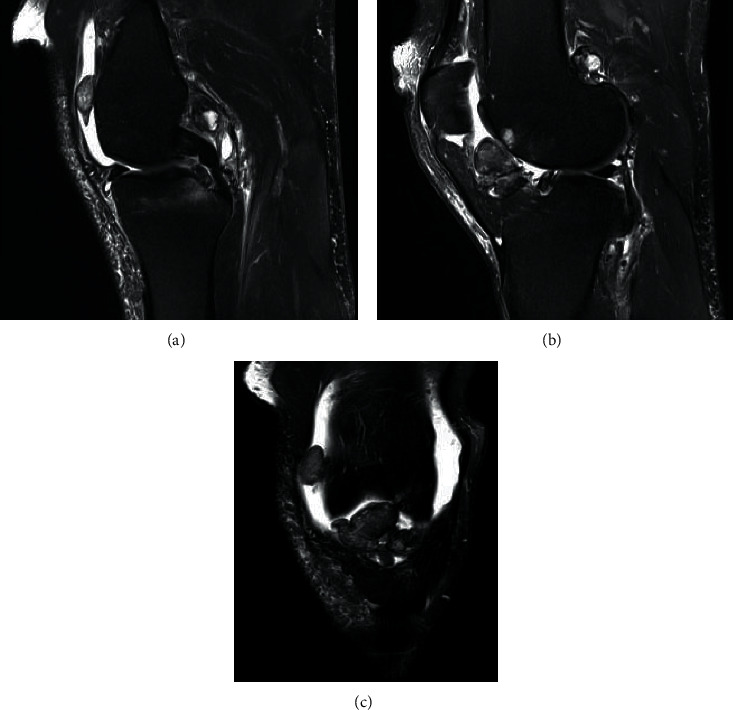
(a) T2 fat-suppressed (FS) (TR/TE: 5070/76) midsagittal view of the left knee. TGCT nodules can be seen in the patellofemoral location and superior to the posterior cruciate ligament. (b) T2 FS (TR/TE: 5070/76) parasagittal view showing TGCT  nodules anterior to the intercondylar notch and posterior-superior to the lateral femoral condyle. (c). T2 FS (TR/TE: 4740/76) coronal view showing the same nodules seen in the patellofemoral region of image (a) and the anterior joint region in image (b). The diffuse low signal of the tumors due to hemosiderin deposition is noted.

Our patient was a seemingly healthy 56-year-old male (1.88 m height and 125 kg (BMI = 33.4)) who was admitted to our hospital for acute left knee pain and swelling. An attorney by profession, he had a medical history of non-insulin-dependent (type 2) diabetes mellitus and asthma. He was not taking any medications for his diabetes and infrequently used a corticosteroid inhaler for asthma symptoms. He had no prior history of knee surgery or prior corticosteroid injections into his left knee. Three months prior to being admitted to our hospital, the patient had seen an orthopaedic surgeon for left knee pain. At that time, magnetic resonance imaging (MRI) was performed of his left knee and revealed several multilobulated masses, which can be seen in Figure 1. All of these lesions showed diffuse abnormal enhancements and low signal on T1- and T2-weighted images, consistent with the presence of hemosiderin deposits of TGCT [7–9]. Other MRI findings included mild-to-moderate osteoarthritis of the patellofemoral articulation and posterior-medial femoral condyle and partial tearing of the medial meniscus. Arthroscopic surgery was recommended, but he delayed this in order to accommodate his work schedule.

While out of town, the patient was admitted to a hospital for possible septic arthritis of the left knee. His synovial fluid cultures showed no growth, and a venous blood sample showed leukocytosis (14.1 K/*μ*L). He was treated with IV antibiotics and instructed to seek continued medical care at his local hospital which was a day's drive away. At our hospital, a second arthrocentesis of his left knee yielded 50 cc of cloudy yellowish fluid. The synovial fluid culture showed no growth, and no crystals were seen under microscopic examination. The knee aspirate cell counts were <3,000 rbc/*μ*L and 30,426 wbc/*μ*L with 90% neutrophils, 8% monocytes, and 2% lymphocytes. His fasting blood glucose was measured at 122 and 184 mg/dL (normal range: 65–99 mg/dL) over the course of his stay. CRP was 11.0 mg/dL (normal range: 0.0–1.0 mg/dL). CBC showed persistent leukocytosis with a WBC of 10.7 K/*μ*L (normal range: 3.6–10.6 K/*μ*L). Urinalysis and erythrocyte sedimentation rate (ESR) were negative as were tests for human immunodeficiency virus (HIV), hepatitis C, *Gonococcus*, and *Chlamydia trachomatis*.

The on-call orthopaedic surgeon (JGS) performed arthroscopic surgery that day for presumed joint sepsis. The degenerative medial meniscus tear was debrided, and all synovium that could be visualized was removed including the D-TGCT lesions. The synovium was mildly erythematous with diffuse brownish pigmentation, which appeared consistent with recurrent hemarthrosis that contributes to the pigmentation of the synovium in TGCT [10–12]. Three ∼2 cc tissue biopsies were taken from the inflamed synovium and villonodular fronds of the larger tumor lesions, which were sent for culture and pathological analysis. The tissues were cultured using chocolate agar, sheep blood agar, and MacConkey agar and subsequently incubated in a thioglycolate broth at 37°C in a CO_2_ atmosphere. Analysis for bacteria species was conducted by MALDI-TOF mass spectrometry. Two of the three specimens tested positive for *Klebsiella oxytoca* (KO) (Gram-negative bacilli), and all three samples tested positive for beta-hemolytic (group B) *Streptococcus agalactiae* (GBS) (Gram-positive cocci). Susceptibility tests revealed that both KO and GBS were susceptible to ceftriaxone. Two grams of ceftriaxone were given IV every 24 hours for four weeks.

**Figure 2:**
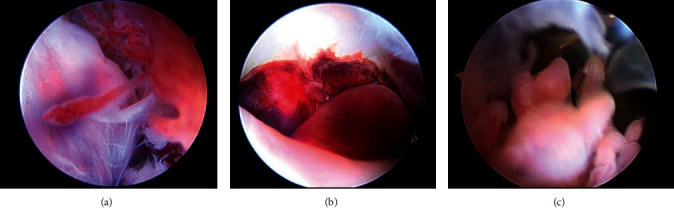
Arthroscopic images showing (a) synovitis that was not believed to have TGCT tissue, (b) infarcted TGCT tissue, and (c) viable TGCT tissue.

**Figure 3:**
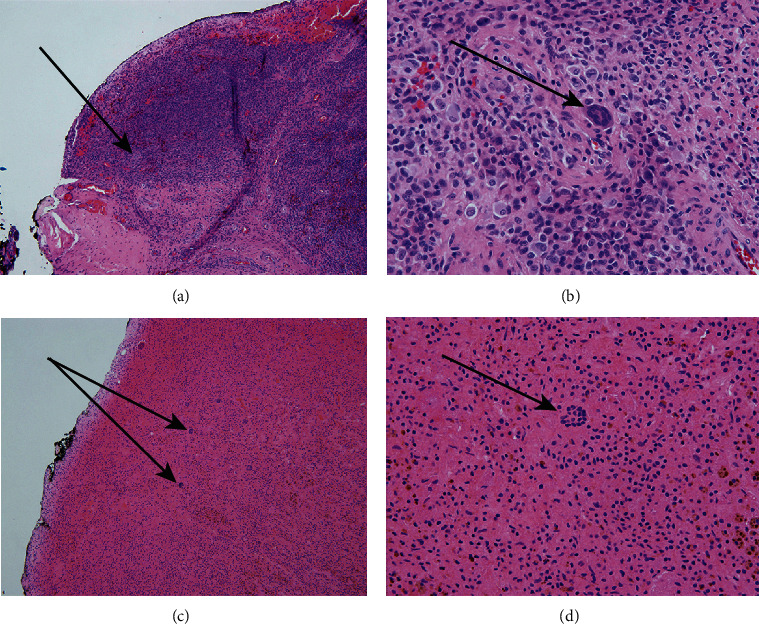
Microscopic images of hematoxylin-eosin-stained sections of tumor tissue. Viable TGCT: (a) 100x image illustrating viable TGCT with scattered giant cells (arrows) and (b) 200x image showing a multinucleated giant cell. Infarcted tissue with scattered giant cells (arrows): (c) 100x image of completely infarcted tissue with multinucleated giant cells and (d) 200x image of partially infarcted tissue with a multinucleated giant cell.

Arthroscopic images are shown in Figure 2, and microscopic images of the tumor biopsies are shown in Figure 3. A pathologist consultant (CFC) suspected that the intraarticular lesions seen with MR imaging were TGCT, but because of substantial tissue infarction, he obtained a second opinion from an out-of-state expert on TGCT at a university hospital. The diagnosis of TGCT was then confirmed after examination of additional tissue sections. One year after surgery, the patient reported that he was “fully recovered” and had no evidence of recurrent infection. He is currently scheduled for a follow-up MR scan to provide a postoperative baseline for future comparative analysis should he become symptomatic.

## 3. Discussion

Infections caused by KO are nearly always nonarticular and predominately occur in the nosocomial setting in patients with concurrent medical conditions [13–18]. Similarly, the likelihood of a GBS infection is increased in individuals who are immunocompromised because of underlying conditions such as diabetes [19–21]. When compared to KO joint infections, GBS joint infections are more common [22–27]. Although we could not locate any reported case of a polymicrobial joint infection with KO with any other bacteria species, polymicrobial joint infections are not rare, accounting for about 10% of nongonococcal cases in adults [26, 28].

Of the nearly one-dozen species within the genus *Klebsiella*, *K. pneumoniae* is considered the most medically important, causing 75% to 86% of clinical *Klebsiella* infections. The second most commonly cultured species is KO, which accounts for 13% to 25% of *Klebsiella* infections [29–32]. Unlike the occasional occurrence of septic arthritis caused by *Klebsiella pneumoniae*, reported cases of bacterial septic arthritis caused by KO are exceedingly rare. In fact, we located only three cases in native (no prior surgery) joints of adults [33].

Given that our patient had no recent hospitalization or invasive medical care, the most likely source of bacterial inoculation was from his antecedent leg laceration from striking it directly on a metal trail hitch. Because normal human skin does not provide good growth conditions for *Klebsiella* species, they are regarded simply as transient members of the skin flora [34, 35]. In contrast, GBS is known to colonize normal skin flora [36]. However, *Klebsiella* and *Streptococcus* species can exist for up to several weeks on inanimate objects, including soil and clothing [37, 38]. The introduction of these pathogens via laceration is supported by several clinical studies of patients with KO or GBS bacteremia [13–15, 21, 23, 39, 40].

The most unusual aspect of our unique case was the concurrence of a bacterial infection within the same joint diseased with D-TGCT. Considering how other proliferative inflammatory diseases such as rheumatoid arthritis increase the likelihood of septic arthritis [26, 41, 42], we considered the possibility that the presence of D-TGCT might influence the risk of hematogenous seeding. While synovial membranes have rich vascularity without a limiting basement membrane [26] and TGCT increases production of extracellular matrix proteins (e.g., fibronectin-, fibrogen-, and laminin-binding proteins) which provide an environment for bacterial adhesion, the connection between TGCT and septic arthritis of this case seems incidental considering there have not been any reported cases of TGCT and septic arthritis. However, while these factors may not inherently increase the risk of septic arthritis, the local environment was such that provided much needed factors for attachment/adhesion, colonization, and persistence for both GBS and KO.

In a comprehensive review of epidemiology, taxonomy, and pathogenicity factors of *Klebsiella* species, [35] discuss how the growth of bacteria in host tissue is limited not only by the host defense mechanisms but also by the supply of available iron. Iron is essential for bacterial growth, and the supply of free iron available to bacteria in the host milieu is extremely low in most cases. Large deposits of iron can occur in the synovium parenchyma of patients with TGCT as the result of repeated bleeding and subsequent hemoglobin degradation. The hemosiderin-laden parenchyma that is characteristic of TGCT is in large part the result of recurrent hemarthrosis and gives TGCT its distinct MRI characteristics [7, 10, 11, 43]. KO, like *K. pneumoniae*, can promote growth and biofilm formation and enhance virulence by chelating iron [44, 45] that is readily available in joints burdened by TGCT.

MRI analysis is considered the most useful presurgical diagnostic modality of TGCT [7, 8, 46]. In our patient, the severity of D-TGCT based on MRI parameters was classified as moderate diffuse according to the study in [8] (i.e., no musculature, tendon, or ligament was involved, and the lesion was confined to the articular capsule of the knee). Based on these criteria, our patient is estimated to have 59% likelihood to be recurrence free 4 years postoperatively [8]. Because there was likely residual synovium posterior to the femoral condyles after arthroscopy, we plan to monitor our patient for recurrence.

## 4. Conclusions

We report the unique case of a patient with polymicrobial bacterial infection and D-TGCT  of the same knee. One of the bacteria species is common (group B *Streptococcus*) in joint sepsis, and the other, *Klebsiella oxytoca*, is extremely uncommon. Characteristics of the synovial membrane and the presence of increased production of fibronectin and other extracellular binding proteins and free iron associated with D-TGCT likely provided an environment that provided a good host for bacterial colonization of his knee. Intravenous antibiotics and arthroscopic debridement effectively treated the infection. During the same arthroscopic surgery, the D-TGCT tissues were excised. Because of the high risk of recurrence, we instructed our patient to follow-up if the knee becomes symptomatic.
